# A Multisystem Perspective of Pediatric Cell Trafficking Disorders: Within the Cells, Beneath the Signs

**DOI:** 10.1002/jmd2.70053

**Published:** 2025-12-28

**Authors:** Merve Yoldaş Çelik, Burcu Köşeci, Ezgi Burgaç, Kanay Yararbaş, Tamer Çelik, Esra Sarıgeçili, Habibe Koç Uçar

**Affiliations:** ^1^ Adana City Training and Research Hospital Department of Pediatric Metabolism Adana Türkiye; ^2^ Sapiens Genetics Laboratory Adana Türkiye; ^3^ Adana City Training and Research Hospital Department of Pediatric Neurology Adana Türkiye

**Keywords:** cellular trafficking disorders, CTD, endoplasmic reticulum dysfunction, Golgi dysfunction, intracellular transport defects

## Abstract

Cell trafficking disorders(CTDs) are rare, heterogeneous inherited conditions marked by impaired intracellular transport mechanisms such as vesicular trafficking, cytoskeletal dynamics, and organelle interactions. Although clinical awareness is increasing, CTDs are often underdiagnosed due to phenotypic overlap with mitochondrial, lysosomal, and glycosylation disorders. We retrospectively analyzed 14 pediatric patients with molecularly confirmed CTDs at a single center. Clinical, biochemical, imaging, and genetic findings were reviewed to explore genotype–phenotype relationships and shared clinical features. The cohort included 8 females and 6 males, with a median diagnostic age of 29 months (range: 1–86 months). Common initial symptoms were developmental delay, hypotonia, seizures, and hepatosplenomegaly. MRI abnormalities were noted in 7 patients. Elevated serum lactate and dicarboxylic aciduria were observed in 9 and 6 patients, respectively. Creatine kinase was raised in several cases, prominently in one with TANGO2 deficiency. Elevated AST (*n* = 12) and ALT (*n* = 5) indicated mild hepatic involvement. Immunological abnormalities included immunoglobulin deficiency (*n* = 3) and protein C/S deficiency (*n* = 4). Recurrently mutated genes were *AP4M1* and *NPC1* (*n* = 2 each); others included *BSCL2*, *PACS1*, *RAB3GAP1*, *STXBP1*, *TANGO2*, *HERC1*, *KIF1A*, *ATP1A3*, *VPS13B*, and *NLGN3*. Two novel variants were identified: AP4M1 c.929 + 1G>T and NPC1 c.145A>T. This case series highlights the clinical and biochemical convergence of genetically diverse CTDs, emphasizing the role of trafficking defects in neurodevelopmental and systemic dysfunction. Expanding diagnostic panels to include trafficking‐related genes and adopting a mechanism‐based classification may improve early recognition and tailored management of these complex disorders.

## Summary

- What is known
  ○ Cell trafficking disorders (CTDs) are a genetically heterogeneous group of IMDs with overlapping features mimicking mitochondrial, lysosomal, and glycosylation disorders.
  ○ CTDs involve diverse defects in intracellular transport mechanisms, including vesicular trafficking, cytoskeletal transport, organelle dynamics, and protein/lipid homeostasis.
- What is new
  ○ This series delineates shared and subtype‐specific features across 14 genetically confirmed CTDs, identifies two novel variants, and supports mechanism‐based diagnostic approaches.

## 1 Introduction

Cell trafficking disorders (CTDs) represent a highly heterogeneous group of inherited metabolic disorders (IMDs) characterized by defects in intracellular transport mechanisms involving vesicles, cytoskeletal components, and organelle contact sites. Although CTDs have been increasingly recognized as distinct clinical entities, they remain underdiagnosed due to their overlapping clinical presentations and multisystem involvement, mimicking mitochondrial, lysosomal, and congenital disorders of glycosylation (CDG) [1].

The expanding spectrum of CTDs now includes mutations in more than 370 genes, encompassing diverse subtypes such as vesicular trafficking defects, axonal transport disorders, and autophagy‐related conditions. Each subtype can present with a wide range of clinical manifestations, including developmental delay, spasticity, seizures, dysmorphic features, and metabolic crises. Given the phenotypic variability and the rarity of each subtype, case series provide a valuable framework for delineating clinical and genetic correlations across different CTD subtypes, enhancing diagnostic accuracy and facilitating early intervention [2].

In this case series, we describe 14 patients with genetically confirmed cellular trafficking disorders, each harboring a distinct pathogenic variant impacting a specific intracellular transport mechanism. The cohort encompasses a diverse range of trafficking subtypes, including vesicular transport (AP4M1, STXBP1), cytoskeletal and axonal transport (KIF1A), lipid metabolism and ER‐Golgi dynamics (BSCL2, PACS1, TANGO2), lysosomal trafficking (NPC1), synaptic vesicle function (NLGN3), and ubiquitin‐mediated regulation (HERC1), thereby enabling a comprehensive exploration of the phenotypic variability and molecular complexity inherent to CTDs. Through systematic evaluation of clinical features, neuroimaging findings, and molecular data, this study seeks to delineate both convergent and subtype‐specific manifestations of CTDs. Our findings underscore the diagnostic challenges posed by these multisystemic disorders and highlight the critical need for an integrated, multidisciplinary approach to their recognition and classification.

## 2 Methods

This study is a retrospective, single‐center case series conducted at a tertiary pediatric metabolism center. The study included 14 patients diagnosed with CTDs. All patients had a confirmed molecular diagnosis via whole‐exome sequencing (WES). Clinical, genetic, and laboratory data were systematically collected and reviewed. Data were extracted from electronic medical records, genetic testing reports, and laboratory databases. The collected data included demographic data (age, sex, consanguinity status, and age at diagnosis), clinical data (presenting complaints, neurological findings, developmental delay, dysmorphic features, and metabolic decompensation), imaging data (Brain MRI findings), genetic data (identified gene mutations, mutation types, inheritance patterns, and ACMG classification), and laboratory data (serum lactate levels, CK levels, liver enzymes, lipid profile, immunoglobulin levels, and lysosomal enzyme activity).

Descriptive statistics were used to summarize clinical and laboratory findings, including frequencies, medians, and ranges. Clinical and genetic findings were stratified by gene subtype to assess genotype–phenotype correlations. Metabolic decompensation, infection susceptibility, and dyslipidemia were specifically analyzed in relation to gene‐specific findings.

The study was conducted in accordance with the principles of the Declaration of Helsinki and received ethical approval from the local ethics committee. Patient confidentiality was maintained throughout the study, and data were anonymized prior to analysis. The study period extended from June 2023 to December 2024. The Institutional Review Board approved the study, and written informed consent was obtained from the patients' legal guardians.

## 3 Results

This study included 14 patients (8 females, 6 males) diagnosed with various subtypes of CTDs. The median age at diagnosis was 29 months (range: 1–86 months), and symptom onset occurred during infancy or early childhood in most cases. The most common presenting complaints were developmental delay, seizures, hypotonia, and hepatosplenomegaly.

Neurological symptoms were observed in nearly all patients (13/14), with muscle tone abnormalities such as spasticity or hypotonia present in 11 patients. Epileptic seizures were reported in 6 patients, and dysmorphic features were documented in 9 cases, including coarse facial features, hypertelorism, high‐arched palate, and micrognathia.

Parental consanguinity was noted in 11 patients, consistent with the high frequency of autosomal recessive inheritance in this cohort. Brain MRI abnormalities were observed in at least 7 patients, including ventricular dilatation, hydrocephalus, cortical atrophy, and periventricular hyperintensities. One patient had dilated cardiomyopathy, and EMG findings revealed myopathy in one case. Metabolic decompensation, including rhabdomyolysis or febrile seizures, was reported in 2 patients. Recurrent infections were noted in 3 cases. The detailed clinical features of all patients are summarized in Table 1.

### 3.1 Genetic Findings

In this cohort, each of the 14 patients was diagnosed with a distinct subtype of cellular trafficking disorder, reflecting the heterogeneity of these conditions. The most frequently affected genes were *NPC1* (*n* = 2) and *AP4M1* (*n* = 2), while all other genes were represented only once.

The most common mutation type was nonsense (*n* = 7), followed by splice‐site (*n* = 4) and missense (*n* = 3) variants. The majority of patients (*n* = 8) carried homozygous mutations, with autosomal recessive inheritance identified in 10 cases. Three patients harbored autosomal dominant variants, and one (*NLGN3*) had an X‐linked mutation.

According to the ACMG classification, 9 variants were deemed pathogenic, 4 likely pathogenic, and 1 was classified as a variant of uncertain significance (VUS). Additionally, two novel variants were identified: *AP4M1* c.929 + 1G>T, affecting a canonical splice donor site, and *NPC1* c.145A>T (p.Asn49Tyr), a rare missense variant not reported in public databases or the literature. The comprehensive genotypic data for each patient are summarized in Table 2.

**TABLE 1:** Clinical, neuroimaging, and systemic findings in CTDs patients.

Patient no	Genetic diagnosis	Sex	Age at diagnosis	Current age	Parental consanguinity	Presenting complaint	Dismorphy	Neurological symptoms
1	BSCL2	F	36	57	Yes	Lipodystrophy, hepatomegaly	Coarse face, large mouth, wide nostrils	+
2	PACS1	F	3	20	Yes	Cholestasis	Microcephaly	+
3	RAB3GA	M	86	100	No	Spasticity, cataract	Joint laxity, crooked teeth	+
4	STXBP1	F	10	31	Yes	Seizures, hyperkplexia	No	+
5	AP4M1	F	16	32	Yes	Hypotonia, seizures	Hypertelorism, broad forehead, micrognathia	+
6	AP4M1	M	26	36	Yes	Hypotonia, seizures	Short neck	+
7	TANGO2	F	47	60	Yes	Encephalopathy, neuroregression, seizures, dystonia, metabolic acidosis	No	+
8	HERC1	F	9	29	Yes	Developmental delay	High palate, increased laxity	+
9	NLGN3	M	56	69	No	Developmental delay	Protruding ears, crooked teeth, high palate	+
10	ATP1A3	F	55	75	No	Developmental delay	No	+
11	KIF1A	M	34	52	Yes	Growth retardation, spastic paraplegia	No	+
12	VPS13B	F	23	28	Yes	Developmental delay	Open mouth, micrognathia, low‐set ears	+
13	NPC1	M	32	44	Yes	Hepatosplenomegaly	Coarse face	+
14	NPC1	M	1	6	Yes	Splenomegaly	No	−

Patient no	Developmental delay	Epilepsy	Muscle tone abnormality	EMG	ECHO	Brain MRI findings	Metabolic decompensation	Infection susceptibility
1	+	−	−	N	N	N	−	−
2	+	−	−	N/A	Secundum ASD	N	−	+
3	+	+	+	N/A	N	Ventricle dilatation, hypomyelination	−	−
4	+	+	+	N/A	N	N	−	−
5	+	+	+	N	N	Hydrocephalus, periventricular hyperintensity	−	−
6	+	+	+	N	N	Periventricular hyperintensity	Febrile convulsions	+
7	+	+	+	myopathy	dilated cardiomyopathy	N/A	Rhabdomyolysis attacks	−
8	+	+	+	N	N	N	−	+
9	+	−	−	N	N	Cyst in right temporal region	−	−
10	+	−	−	N	N	N	−	−
11	+	−	+	N	N	Ventricular asymmetry	−	−
12	+	−	+	N	N/A	Bilateral temporal sulcal widening	−	−
13	+	−	+	N	N	Progressive white matter changes	−	−
14	−	−	−	N	N	N/A	−	−

Abbreviations: ECHO: echocardiography; EMG: electromyography; MRI: magnetic resonance imaging; N/A: not available; N: normal.

**TABLE 2:** Summary of genetic variants and pathogenetic classification in CTDs.

Patient no	Gene	CTD subtype	Pathogenetic mechanism	Nucleotide change	Protein change	Mutation type	Zygosity	Inheritance pattern	Pathogenicity
1	*BSCL2*	ER‐related disorder	Seipin deficiency disrupts lipid droplet formation and ER membrane homeostasis.	c.631del	p.Val211*	Nonsense	Homozygous	Autosomal recessive	Pathogenic
2	*PACS1*	Golgipathy	Impairs Golgi‐to‐nucleus protein trafficking and vesicle sorting.	c.1471C>T	p.Gln491*	Nonsense	Heterozygous	Autosomal dominant	Likely pathogenic
3	*RAB3GAP1*	Rabopathy/golgi trafficking	Defective Rab‐GTPase regulation affects ER‐to‐Golgi vesicle targeting.	c.2709 + 1G>T	—	Splice‐site	Homozygous	Autosomal recessive	Likely pathogenic
4	*STXBP1*	SNAREopathy	Disruption of synaptic vesicle fusion at presynaptic terminals.	c.1099C>T	p.Arg367*	Nonsense	Heterozygous	Autosomal dominant	Pathogenic
5	*AP4M1*	Adaptinopathy	Loss of AP‐4 adaptor impairs cargo sorting at trans‐Golgi network.	c. 1012C>T	p.Arg338*	Nonsense	Homozygous	Autosomal recessive	Pathogenic
6	*AP4M1*	Adaptinopathy	Loss of AP‐4 adaptor impairs cargo sorting at trans‐Golgi network.	c.929 + 1G>T	—	Splice‐site	Homozygous	Autosomal recessive	Pathogenic
7	*TANGO2*	ER‐mitochondrial transport	Defects at ER‐mitochondrial contact sites disrupt lipid and energy homeostasis.	c.145 + 1G>T	—	Splice‐site	Homozygous	Autosomal recessive	Likely pathogenic
8	*HERC1*	Ubiquitin trafficking disorder	Ubiquitin ligase dysfunction impairs endosomal sorting and membrane turnover.	c.4463 + 1G>A	—	Splice‐site	Homozygous	Autosomal recessive	Pathogenic
9	*NLGN3*	Synaptopathy	Disrupts postsynaptic membrane formation and synaptic signaling.	c.43A>G	p.Lys15Glu	Missense	Hemizygous	X‐linked	VUS
10	*ATP1A3*	Flippase‐related disorder/ion transport disorder	Aberrant Na^+^/K^+^ ATPase activity affects membrane potential and trafficking.	c.2438C>T	p.Ala813Val	Missense	Heterozygous	Autosomal dominant	Likely pathogenic
11	*KIF1A*	Kinesinopathy	Kinesin‐3 mutations impair anterograde vesicle/organelle transport.	c.31C>T	p.Arg11Trp	Missense	Heterozygous	Autosomal dominant	Pathogenic
12	*VPS13B*	Golgipathy	Affects vesicle budding and cargo delivery via Golgi membrane contact sites.	c.8827C>T	p.Arg2943Ter	Nonsense	Homozygous	Autosomal recessive	Pathogenic
13	*NPC1*	Lysosomal trafficking disorder	Blocks egress of cholesterol from late endosomes and lysosomes.	c.145A>T	p.K49*	Nonsense	Homozygous	Autosomal recessive	Likely pathogenic
14	*NPC1*	Lysosomal trafficking disorder	Blocks egress of cholesterol from late endosomes and lysosomes.	c.145A>T	p.K49*	Nonsense	Homozygous	Autosomal recessive	Likely pathogenic

*Note:* Pathogenicity classification is based on ACMG guidelines, incorporating variant type, inheritance pattern, and reported clinical relevance.

**TABLE 3:** Biochemical and immunological profiles of the CTDs cohort.

Patient no	Genetic diagnosis	Lipid metabolism				Liver and muscle enzymes			Immun system				Lysosomal enzyme activity	Additionally
		Triglyceride (mg/dL)	LDL (mg/dL)	HDL (mg/dL)	Total cholesterol (mg/dL)	CK (U/L)	AST (U/L)	ALT (U/L)	IgG	IgA	IgM	Neutrophil count (/μL)		
1	BSCL2	1312	77	18	127	63	41	65	N	Low	N	2880	N	High insulin levels
2	PACS1	295	290	37	380	37	229	184	Low	Low	Low	3700	N	
3	RAB3GA	N/A	N/A	N/A	N/A	43	34	44	N/A	N/A	N/A	5500	N	
4	STXBP1	N/A	N/A	N/A	N/A	181	43	11	N/A	N/A	N/A	3200	N	Low protein S: % 36.9 (NR 60–150)
5	AP4M1	96	96	39	137	629	43	11	N	N	N	5050	N	Low protein C: %63 (NR 70–140), Low protein S: % 50 (NR 60–150)
6	AP4M1	230	85	41	139	174	28	13	N	N	N	5000	N	
7	TANGO2	140	70	37	123	1902	102	123	N	N	N	5200	N	Hypothyroidism
8	HERC1	131	88	45	146	241	46	27	N	N	N	1500	Low	Low protein C: %35 (NR 70–140)
9	NLGN3	N/A	N/A	N/A	N/A	188	55	18	N	N	N	4400	N	
10	ATP1A3	94	99	49	157	120	45	40	N/A	N/A	N/A	3100	N	
11	KIF1A	72	118	59	189	89	55	15	N	Low	N	2000	N	Low protein C: %69 (NR 70–140), Low protein S: % 41 (NR 60–150)
12	VPS13B	142	91	55	151	181	49	22	N	N	N	300	N	
13	NPC1	100	43	22	73	17	56	9	N	N	N	2160	N	
14	NPC1	182	72	65	134	105	55	35	N	N	N	6510	N	

*Note:* Reference ranges: Triglyceride < 150 mg/dL; Low‐density lipoprotein (LDL) < 130 mg/dL; High‐density lipoprotein (HDL) > 40 mg/dL; Total cholesterol < 200 mg/dL; Creatine kinase (CK) < 171 U/L; Aspartate aminotransferase (AST) < 40 U/L; Alanine aminotransferase (ALT) < 40 U/L; Absolute neutrophil count > 1500/μL.

Abbreviations: Ig: Immunoglobulin; N/A: not available; N: normal; NR, normal range.

**TABLE 4:** Biochemical parameters related to mitochondrial function and energy metabolism in patients with cell‐trafficking disorders.

No	Genetic diagnosis	Lactate (mg/dL, NR 5–22)	Pyruvate (mg/dL, NR 0.3–0.9)	Acid–base status (pH HCO_3_^−^ mmol/L)	Alanine (μmol/L, NR 160–530)	Glutamine (μmol/L, NR 380–680)	Proline (μmol/L, NR 90–350)	Glycine (μmol/L, NR 140–420)	Serine (μmol/L, NR 60–170)	Dicarboxylic aciduria (urine organic acids)	Quantified acids/profile	Interpretation
1	BSCL2	10–17	N/A	N/A	382	N/A	N/A	N/A	N/A	−	N/A	−
2	PACS1	29–40 ↑	0.71	7.38/21	375	176 ↓	192	291	154	+	Suberic acid 22.78 mmol/mol creatinine (Ref 0–12)	Mild biochemical changes (↑lactate, ↓glutamine) consistent with possible secondary mitochondrial dysfunction.
3	RAB3GA	20–29 ↑	0.75	7.39/22	N/A	N/A	N/A	N/A	N/A	−	N/A	−
4	STXBP1	11–20	0.32	7.42/19	233	445	121	148	78	+	Ethylmalonic acid 17.8 (Ref 0–17); Methylsuccinic acid 7.29 (Ref 0–6); 3‐Methylglutaconic acid 13.01 (Ref 0–9)	Dicarboxylic aciduria pattern *suggestive of* secondary β‐oxidation disturbance with compensatory ω‐oxidation, *possibly reflecting ER–mitochondrial stress*.
5	AP4M1	25–27 ↑	0.56	7.43/25	602 ↑	498	165	279	221 ↑	−	N/A	Isolated alanine elevation indicating mild redox imbalance; otherwise unremarkable metabolic profile.
6	AP4M1	10–14	N/A	7.34/21	334	528	259	118 ↓	84	−	N/A	No clear evidence of mitochondrial involvement.
7	TANGO2	27 ↑	3.0 ↑	7.42/23	304	288	196	109 ↓	46 ↓	+	Fumaric acid 37.34 (Ref 0–17); Glutaric acid 39.58 (Ref 0–8); Adipic acid 1432.48 (Ref 0–20); Suberic acid 690.38 (Ref 0–12); Sebacic acid 253.58 (Ref 0–2)	Biochemical profile *compatible with* mitochondrial energy stress and compensatory ω‐oxidation.
8	HERC1	11–26 ↑	0.73	7.41/22	235	444	310	149	117	+	Adipic acid 22.91 (Ref 0–20); 3‐Methylglutaconic acid 12.36 (Ref 0–9)	Subtle organic‐acid changes (*adipic* and *3‐methylglutaconic*) consistent with mild secondary mitochondrial stress.
9	NLGN3	22–34 ↑	1.38 ↑	7.39/21	432	732	155	376	83	+	Tiglylglycine 4.79 (Ref 0–2)	Minor, nonspecific metabolic alterations (*pyruvate*, *glutamine*), clinical relevance uncertain.
10	ATP1A3	18–28 ↑	0.9	7.40/20	535 ↑	667	353 ↑	492 ↑	206 ↑	−	N/A	Broad amino‐acid elevations (*alanine*, *proline*, *glycine*, *serine*) possibly reflecting nonspecific catabolic or redox stress.
11	KIF1A	25–29 ↑	1.0 ↑	7.38/22	164	472	83 ↓	175	84	−	N/A	Largely normal metabolic profile (isolated low proline without functional significance)
12	VPS13B	12–22	N/A	7.46/25	361	504	208	253	217 ↑	−	N/A	Biochemical findings within reference range; no apparent mitochondrial disturbance
13	NPC1	12–31 ↑	0.8	7.41/23	460	593	295	462 ↑	131	+	Adipic acid 57.48 mmol/mol creatinine (Ref 0–20)	Dicarboxylic aciduria with elevated alanine, glutamine, and glycine, *consistent with* secondary mitochondrial involvement due to ER–mitochondrial crosstalk stress.
14	NPC1	8–10	N/A	7.44/24	N/A	N/A	N/A	N/A	N/A	−	N/A	−

*Note:* Lactate values represent minimum–maximum ranges across repeated measurements when available. Interpretations are based on integrated assessment of lactate, amino acid, and organic acid profiles to evaluate secondary mitochondrial dysfunction potentially related to organelle trafficking defects.

Abbreviations: AA, amino acid; FAO, fatty acid oxidation; ER, endoplasmic reticulum; NR, normal range; N/A, not available; ↑, above reference range; ↓, below reference range; ω‐oxidation, omega oxidation pathway.

### 3.2 Laboratory Findings

Laboratory investigations revealed a heterogeneous biochemical profile across patients. Creatine kinase (CK) levels were elevated in several patients, with values exceeding 1000 U/L only in the patient with TANGO2‐related metabolic encephalopathy. Elevated AST levels were observed in 12 patients and ALT elevation in 5 patients (defined as > 40 U/L), indicating mild hepatic involvement in a majority of cases. Dyslipidemia was observed in several patients, including elevated triglycerides (> 150 mg/dL) in 4 cases, low HDL levels (< 40 mg/dL) in 5, and high LDL or total cholesterol in 1 patient each. The most pronounced dyslipidemic profiles were noted in patients with BSCL2‐ and PACS1‐related disorders.

Regarding immune parameters, 3 patients had low immunoglobulin levels (IgG, IgA, and/or IgM), and two patients had neutropenia. Lysosomal enzyme activity was generally within the normal range, except in the HERC1 patient. Additional findings included protein C and/or S deficiencies in 4 patients, hypothyroidism in one (TANGO2), and hyperinsulinemia in the BSCL2 patient. A detailed summary of laboratory parameters, categorized by system, is shown in Table 3.

Elevated lactate concentrations were observed in nine patients, ranging from mild to moderate levels. Metabolic acidosis was not detected in any case. Alanine was above the reference range in two patients, while other amino acids such as glutamine and proline showed mild or inconsistent deviations. Dicarboxylic aciduria was present in six patients, with adipic, suberic, sebacic, or fumaric acids being the most frequent findings. Parameters related to mitochondrial and energy metabolism, including lactate, pyruvate, amino‐acid profiles, and organic‐acid analysis, are summarized in Table 4.

## 4 Case Descriptions

The following case‐by‐case summaries provide a concise clinical and molecular overview of the 14 patients included in this study. Each case is categorized according to the underlying genetic diagnosis and its corresponding subtype within the spectrum of CTDs.

### 4.1 Case 1: BSCL2‐Related Congenital Lipodystrophy

A 3‐year‐old female patient presented with developmental delay, dysmorphic features, and lipodystrophy. Genetic analysis revealed a homozygous c.631del (p.Val211*) pathogenic variant in the *BSCL2* gene. Clinical findings included hepatosteatosis, eruptive xanthomas, dysphagia, and spastic gait. Imaging demonstrated hepatomegaly and splenomegaly with increased liver parenchymal echogenicity. Neurological examination showed a broad‐based, slightly ataxic gait and hypotonia.

### 4.2 Case 2: PACS1‐Related Schuurs–Hoeijmakers Syndrome

A 3‐month‐old female infant was referred for evaluation of cholestasis. Genetic testing identified a heterozygous c.1471C>T (p.Gln491*) variant in the *PACS1* gene, associated with Schuurs‐Hoeijmakers syndrome. Clinical evaluation revealed developmental delay, hepatomegaly, and persistent hyperbilirubinemia. Hepatic biopsy demonstrated cholestatic parenchymal damage with giant cell transformation. Further analysis included comprehensive metabolic testing, which was unremarkable.

### 4.3 Case 3: RAB3GAP1‐Related Warburg Micro Syndrome

A 4‐year‐old male patient diagnosed with Warburg Micro Syndrome based on a homozygous c.2709 + 1G>T pathogenic splice site variant in the *RAB3GAP1* gene. The patient exhibited severe neurodevelopmental delay, spasticity, cataract, and dysmorphic features. MRI revealed ventriculomegaly, thinning of the corpus callosum, and gliosis in the periventricular white matter. Skeletal deformities included joint contractures and lower limb spasticity.

### 4.4 Case 4: STXBP1‐Associated Epileptic Encephalopathy

A 19‐month‐old female patient presented with recurrent seizures and profound developmental delay. Genetic analysis revealed a heterozygous c.1099C>T (p.Arg367*) pathogenic variant in the *STXBP1* gene, leading to impaired synaptic vesicle fusion. Despite treatment with antiepileptic drugs, the patient continues to experience tremors and motor delays. Imaging studies were unremarkable, and electrophysiological testing confirmed generalized epileptic activity.

### 4.5 Case 5: AP4M1‐Related Spastic Paraplegia

A 2‐year‐old female patient with spastic paraplegia and developmental delay was diagnosed with an AP4M1‐related adaptinopathy based on a homozygous c.1012C>T (p.Arg338*) variant. MRI revealed ventricular enlargement, corpus callosum thinning, and periventricular white matter gliosis. The clinical presentation was consistent with early‐onset spastic paraplegia and motor coordination deficits, with no reported seizures.

### 4.6 Case 6: AP4M1‐Related Spastic Paraplegia

A 1‐year‐old male patient with recurrent febrile seizures and developmental delay was diagnosed with an AP4M1‐related adaptinopathy due to a homozygous c.929 + 1G>T splice site variant. Family history was notable for multiple relatives with severe neurological impairments. Imaging findings included ventricular asymmetry, cortical thinning, and periventricular white matter changes. The patient demonstrates global hypotonia and motor coordination deficits.

### 4.7 Case 7: TANGO2‐Related Metabolic Encephalopathy

A 3‐year‐11‐month‐old girl, initially presented with episodic encephalopathy, recurrent vomiting, and metabolic acidosis, accompanied by progressive neuroregression and dystonia. Clinical evaluation revealed hypotonia, developmental regression, and episodic metabolic crises characterized by hypoglycemia, elevated lactate, and CK levels. Cardiac evaluation demonstrated borderline systolic dysfunction and dilated cardiomyopathy. The genetic analysis identified a homozygous c.145 + 1G>T splice‐site variant in the *TANGO2* gene, consistent with the lipid trafficking disorder phenotype. Management includes metabolic crisis prevention, cardiac monitoring, supportive care for dystonia and developmental delay, and administration of vitamin supplementation including riboflavin, thiamin, B5, coenzyme Q10, and biotin to support mitochondrial function.

### 4.8 Case 8: HERC1‐Related Neurodevelopmental Disorder

A 12‐month‐old female with dysmorphic features, and severe developmental delay. Genetic testing identified a homozygous c.4463 + 1G>A variant in the *HERC1* gene. Clinical findings included prominent forehead, joint laxity, and hypotonia. MRI findings were unremarkable, but metabolic screening revealed persistent 3‐methylglutaconic aciduria.

### 4.9 Case 9: NLGN3‐Related Neurodevelopmental Disorder

A 4‐year‐old male with intellectual disability, speech delay, and behavioral abnormalities. Whole‐exome sequencing revealed a hemizygous *NLGN3* c.43A>G variant, classified as a variant of uncertain significance (VUS). Parental testing confirmed the variant to be de novo, providing supportive but not conclusive evidence regarding its clinical relevance. Clinical findings included dysmorphic facial features, hypotonia, and mild spasticity. MRI findings showed a small choroid fissure cyst in the right temporal lobe.

### 4.10 Case 10: ATP1A3‐Related Early‐Onset Encephalopathy

A 4‐year‐7‐month‐old girl presented with developmental delay and atypical autism‐like features, including transient episodes of blank staring and hypotonia that resolved within a year. Over time, she exhibited persistent motor delays, generalized hypotonia, and elevated plasma amino acids, particularly alanine, serine, and glycine. Brain MRI and MR spectroscopy revealed no significant structural abnormalities. Comprehensive metabolic workup, including mitochondrial DNA analysis, was unremarkable. Genetic analysis identified a heterozygous c.2438C>T missense variant in the *ATP1A3* gene, associated with CAPOS syndrome, alternating hemiplegia of childhood, and developmental and epileptic encephalopathy.

### 4.11 Case 11: KIF1A‐Related NESCAV Syndrome

A 2‐year‐old male patient with spastic gait, developmental delay, and dystonia. Genetic analysis identified a heterozygous c.31C>T (p.Arg11Trp) pathogenic variant in the *KIF1A* gene, consistent with NESCAV syndrome and spastic paraplegia. MRI findings included lateral ventricular asymmetry and white matter volume loss. The patient has no history of seizures but demonstrates prominent spasticity and ataxia.

### 4.12 Case 12: VPS13B‐Related Cohen Syndrome

A 2‐year‐old girl with a confirmed diagnosis of Cohen syndrome caused by a homozygous c.8827C>T (p.Arg2943Ter) pathogenic variant in the *VPS13B* gene presented with global developmental delay, hypotonia, and speech delay, alongside significant findings of neutropenia and retinal dystrophy. Brain MRI demonstrated bilateral temporal sulcus prominence with otherwise normal findings. Metabolic workup revealed non‐specific amino acid abnormalities. Management focuses on supportive therapies, hematology follow‐up for neutropenia, and multidisciplinary developmental assessment.

### 4.13 Case 13 and 14: NPC1‐Related Niemann‐Pick Disease Type C

Two siblings were diagnosed with Niemann‐Pick disease type C due to a homozygous c.145A>T (p.K49*) variant in the *NPC1* gene. The elder brother, currently 3 years and 8 months old, initially presented with hepatosplenomegaly, anemia, and thrombocytopenia. Clinical regression included loss of previously acquired motor milestones such as sitting and standing. Brain MRI revealed progressive white matter changes, and the patient was initiated on miglustat therapy alongside a ketogenic diet. The younger brother, now 6 months old, was diagnosed prenatally following genetic testing and currently shows no neurological symptoms but is under close monitoring.

## 5 Discussion

CTDs represent a highly heterogeneous group of genetic diseases caused by defects in intracellular transport mechanisms involving vesicles, organelles, cytoskeletal dynamics, and lipid metabolism [3]. Although awareness of CTDs has increased in recent years, these disorders remain significantly underdiagnosed due to their broad phenotypic overlap with mitochondrial diseases, lysosomal storage disorders, and congenital disorders of glycosylation. Even when recognized, CTDs are often classified solely based on clinical phenotypes, while the underlying pathogenic mechanisms, such as defects in membrane trafficking or cytoskeletal transport, remain underappreciated. A pathogenesis‐oriented diagnostic framework could improve classification, management, and therapeutic strategies for these complex disorders. The diversity in genetic etiology leads to a wide range of clinical manifestations, including neurodevelopmental delay, epilepsy, metabolic crises, and multisystemic involvement, further complicating the diagnostic process [2]. In this case series, we present 14 pediatric patients with molecularly confirmed CTDs, spanning 12 different genetic etiologies, including mutations in *BSCL2*, *PACS1*, *RAB3GAP1*, *STXBP1*, *AP4M1*, *HERC1*, *KIF1A*, *ATP1A3*, *NLGN3*, *VPS13B*, and *NPC1*. By integrating clinical, radiological, biochemical, and genetic data, this study provides a detailed characterization of CTDs and underscores their clinical relevance beyond conventional diagnostic categories. The cohort illustrates not only the broad phenotypic variability but also the shared pathophysiological features that emerge across genetically distinct subtypes. These findings suggest that CTDs may be more effectively understood when grouped by underlying molecular mechanisms rather than by isolated clinical features. This perspective aligns with the view proposed by García‐Cazorla et al. who suggest that certain genetic conditions, although not historically classified as IMDs, may involve underlying biochemical disturbances that warrant consideration within the broader framework of IMDs [4].

The clinical spectrum of CTDs is markedly heterogeneous, encompassing a wide array of neurodevelopmental, neuromuscular, and systemic features. In our cohort, developmental delay was the most frequent initial symptom, observed across nearly all genotypes. Muscle tone abnormalities (particularly spasticity and hypotonia) were prominent in cases with *AP4M1* and *KIF1A* mutations, supporting prior evidence linking vesicular and axonal transport disruption to motor pathway involvement. For instance, two patients harboring biallelic *AP4M1* pathogenic variants exemplified the classical phenotype of AP‐4 adaptinopathy, with early‐onset spastic paraplegia, ventriculomegaly, and corpus callosum thinning [5]. One of these patients also exhibited febrile seizures, which have been increasingly recognized in the AP4M1‐related spectrum and may be related to impaired synaptic vesicle cycling and neuroinflammation [6]. Similarly, our KIF1A case demonstrated progressive spastic paraplegia and significant developmental delay, along with neuroimaging findings of ventricular asymmetry and cortical atrophy, consistent with the known impact of *KIF1A* mutations on anterograde axonal transport mediated by kinesin‐3 motor proteins [7, 8, 9]. Additional features such as epileptic encephalopathy (STXBP1), neuroregression, recurrent rhabdomyolysis, and cardiac involvement (TANGO2), and hepatomegaly (NPC1, BSCL2) illustrate the multisystemic nature of CTDs. Brain MRI abnormalities, though variable, were present in the majority of patients, ranging from nonspecific periventricular white matter hyperintensities to structural malformations such as callosal hypoplasia. These findings highlight the importance of recognizing CTDs as neurodevelopmental syndromes with potential involvement of both central and peripheral systems, and underscore the diagnostic utility of combining clinical, radiological, and molecular data.

Laboratory investigations in our CTD cohort showed a heterogeneous but informative biochemical profile. Elevated serum lactate was observed in nine of fourteen patients, and dicarboxylic aciduria in six. These findings form a recurrent metabolic pattern that may reflect subtle alterations in organelle communication. Recent studies have highlighted that physical and functional interactions between the ER and mitochondria play key roles in lipid transfer, calcium homeostasis, and redox regulation [10, 11, 12]. Disruption of these ER–mitochondrial contact sites (MAMs) has been linked to decreased mitochondrial efficiency and increased susceptibility to metabolic stress [13]. Under such conditions, a compensatory shift from β‐oxidation toward ω‐oxidation may occur, resulting in the accumulation of dicarboxylic acids such as adipic, suberic, or sebacic acid [14]. In our patients, the coexistence of mild hyperlactatemia and this organic acid pattern may therefore indicate a secondary metabolic adaptation to organelle‐trafficking dysfunction rather than a primary defect of mitochondrial energy metabolism.

Amino acid profiling in our cohort revealed subtle yet recurrent deviations that may reflect mild metabolic adjustments in response to cellular stress. Slight alanine elevations in some patients could be related to altered pyruvate–lactate dynamics or redox imbalance, whereas variable glutamine levels might suggest changes in anaplerotic support to the TCA cycle [15]. Modest variations in proline and glycine could represent nonspecific adaptive responses linked to redox buffering or glutathione turnover [16]. Occasional decreases in serine may be consistent with enhanced one‐carbon or NADPH‐dependent pathways during oxidative stress [17]. Overall, these patterns may indicate mild and heterogeneous energy–redox adaptations secondary to organelle trafficking disturbances, without implying a primary mitochondrial disorder.

Interestingly, a recent study demonstrated that TANGO2 deficiency can directly impair mitochondrial oxidative metabolism, showing reduced oxygen consumption and β‐oxidation capacity in patient‐derived fibroblasts [18]. This observation supports the possibility that trafficking‐related defects may contribute to variable degrees of mitochondrial vulnerability across the CTD spectrum. Consistent with this observation, our patient with TANGO2‐related disease showed biochemical evidence of energy failure. Markedly increased CK levels exceeding 1000 U/L in the TANGO2 case further support the hypothesis of energy failure and muscular involvement in trafficking‐related disorders, a phenomenon increasingly acknowledged in recent literature, particularly for defects affecting ER‐mitochondria communication [18].

Abnormal transaminase levels were frequently observed in our cohort, with ALT elevation in eight and AST in seven patients, indicating hepatic involvement. Similar hepatic enzyme disturbances have been described in vesicular trafficking disorders such as NBAS, RINT1, and SCYL1 deficiencies, where recurrent episodes of acute liver dysfunction are attributed to impaired ER–Golgi transport and hepatocellular stress [19]. These observations are consistent with the multisystemic nature of cell trafficking disorders, in which hepatocellular vulnerability may accompany neurological and metabolic manifestations due to the shared dependency of multiple organ systems on efficient vesicular and membrane transport.

Regarding lipid metabolism, dyslipidaemia was particularly pronounced in the patient with BSCL2‐related disease, consistent with the established role of seipin in lipid droplet biogenesis and hepatic lipid turnover [20]. In contrast, lipid abnormalities were absent or mild in other CTD subtypes, suggesting that these disturbances are not universal but may arise as secondary manifestations of altered intracellular trafficking or metabolic stress.

Immune system anomalies such as hypogammaglobulinemia and neutropenia were also documented, echoing recent data that CTDs may interfere with membrane‐bound immunoreceptor processing and trafficking [2, 21]. Hypothyroidism and hyperinsulinemia were observed in a subset of patients, suggesting that CTDs may also interfere with endocrine regulation, possibly through altered trafficking of hormonal receptors or secretory granules [22]. The Golgi apparatus and its associated vesicular network are central to protein processing and secretion, and disruption at this level may lead to dysregulated hormone synthesis or release.

Mild reductions in protein C and/or protein S were detected in several patients without any thrombotic manifestations. These alterations may reflect secondary effects of hepatic stress or impaired glycosylation of coagulation factors, as described in congenital disorders of glycosylation [23, 24]. Such findings suggest a potential link between vesicular trafficking, post‐translational protein modification, and hemostatic homeostasis rather than a primary coagulation defect. These findings reinforce the emerging concept that CTDs are not confined to structural neurodevelopmental defects, but also include functional impairments of mitochondrial bioenergetics, hepatic metabolism, immunoregulation, and endocrine signaling, consistent with the multisystemic nature of cell trafficking disruption. However, the biomarkers identified in this series should be interpreted with caution, as they correspond to a small number of patients who are heterogeneous with respect to the genes involved. Studies involving larger cohorts of similar cases are needed to strengthen the validity of these findings.

The genetic heterogeneity observed in this CTD cohort underscores the complexity of intracellular transport pathways and their diverse clinical consequences. While a recurrent variant in A*P4M1* confirmed previously established genotype–phenotype correlations, one of our AP4M1 cases harbored a homozygous c.929 + 1G>T splice‐site variant that, to our knowledge, has not been previously reported. Similarly, both of our NPC1 cases involved a homozygous c.145A>T (p.K49*) nonsense variant, also novel, yet clinically consistent with Niemann‐Pick type C, including hepatosplenomegaly, neuroregression, and progressive white matter changes [25]. The NLGN3 c.43A>G variant, identified as de novo and currently classified as a variant of uncertain significance, was reported descriptively. The patient's developmental and behavioral profile shares partial overlap with previously described NLGN3‐related phenotypes [26]; however, causality cannot be inferred. Functional studies would be required to determine its impact, and the absence of such data represents a limitation of this study.

Collectively, the variants identified in this cohort contribute to the expanding mutational spectrum of trafficking‐related genes, potentially providing insight into their functional diversity. A notable portion of the cohort involved less frequently reported genes such as *BSCL2*, *PACS1*, and *HERC1*, for which trafficking‐related mechanisms are increasingly recognized. In our BSCL2 case, the patient exhibited congenital generalized lipodystrophy and hepatomegaly, along with developmental delay and a normal EMG. Notably, pathogenic variants in *BSCL2* have been associated with markedly distinct phenotypes depending on the mode of inheritance: autosomal dominant variants cause motor neuron disorders such as Silver syndrome and distal hereditary motor neuropathy [27], whereas autosomal recessive mutations lead to generalized lipodystrophy, sometimes accompanied by progressive encephalopathy [28, 29]. The stark contrast between dominant and recessive BSCL2‐related disorders highlights the functional complexity of seipin in both lipid homeostasis and neuronal maintenance, suggesting pleiotropic roles modulated by allelic context and tissue‐specific expression [30]. These findings support the inclusion of emerging CTD‐related genes in diagnostic panels and highlight the need for pathomechanism‐oriented reclassification of disorders historically categorized under broader neurodevelopmental or metabolic umbrellas [2]. As intracellular trafficking defects span multiple organelle systems, this genetic diversity demands a systems biology perspective in both research and clinical genomics [21].

The findings of this study underscore the necessity of re‐evaluating current diagnostic frameworks for early‐onset multisystem disorders. Rather than relying solely on dominant clinical features such as seizures, hypotonia, or hepatosplenomegaly, a nosological approach grounded in molecular pathogenesis may offer improved diagnostic precision and better reflect the biological continuum of CTDs. Conceptualizing CTDs through the lens of shared mechanistic disruptions, such as vesicular transport failure, cytoskeletal instability, and impaired organelle dynamics, may facilitate earlier recognition, more accurate prognostication, and the development of mechanism‐informed therapeutic strategies.

Despite the genetic heterogeneity of CTDs, our cohort revealed converging phenotypic signatures, including spasticity, epilepsy, and neuroimaging abnormalities, which reflect the functional convergence of diverse pathogenic variants on common intracellular pathways. Biochemical findings such as elevated lactate and dyslipidemia further illustrate the systemic metabolic consequences of trafficking dysfunction, particularly in mitochondrial and lipid‐related compartments.

Although constrained by its retrospective nature and limited sample size, this case series provides valuable insights into the expanding genotypic and phenotypic spectrum of CTDs. The identification of rare and novel variants contributes to the refinement of gene‐disease associations and highlights the importance of integrating molecular diagnostics with clinical pattern recognition in the evaluation of complex neurogenetic conditions.

## Author Contributions

Merve Yoldaş Çelik designed the study; Merve Yoldaş Çelik, Burcu Köşeci, Ezgi Burgaç, Kanay Yararbaş, Esra Sarıgeçili, Tamer Çelik, Habibe Koç Uçar collected data; Merve Yoldaş Çelik analyzed data and wrote the manuscript. All authors have accepted responsibility for the entire content of this manuscript and approved its submission.

## Funding

The authors have nothing to report.

## Ethics Statement

This study was performed in line with the principles of the Declaration of Helsinki. Approval was granted by the Ethics Committee of Adana City Training and Research Hospital (Meeting number 9; Date 02/01/2025; Decision number 307).

## Consent

Informed consent was obtained from the parents of the patients in this study.

## Conflicts of Interest

The authors declare no conflicts of interest.

## Data Availability

The data that support the findings of this study are available from the corresponding author upon reasonable request.
